# Electronic Structure of Dinuclear Gold(I) Complexes

**DOI:** 10.1155/MBD.1999.255

**Published:** 1999

**Authors:** Janet B. Foley, Stanley E. Gay, Christopher Turmel, Gang Wei, Tong Jiang, Ratnavathany Narayanaswamy, Bruce M. Foxman, Michael J. Vela, Alice E. Bruce, Mitchell R. M. Bruce

**Affiliations:** 1 University of Maine Department.of Chemistry Orono Maine 04469-5706 USA; 2 Brandeis University Waltham MA 02454-9110 USA

## Abstract

Cyclic voltammetry (CV) experiments on LL(AuSR∗)_2_ complexes [LL = diphenylphosphinomethane (dppm), diphenylphosphinopentane (dpppn); R^*^ = *p*-SC_6_H_4_CH_3_] show anodic sweeps that broaden by about 25 mV on going from the longer (dpppn) to the shorter (dppm) bidentate phosphine ligand. Changing concentrations had no effect on the shape of the waveform. The result suggests a weak intramolecular metal-metal interaction in dppm(AuSR∗)_2_ that correlates well with rate acceleration occurring in the reaction of dppm(AuSR∗)_2_ with organic disulfides. Quantum yields for *cis*-dppee(AuX)_2_ [dppee = 1,2-bis(diphenylphosphino)ethylene; X = Cl, Br, I] complexes, (disappearance) Φ , are significantly higher in complexes with a softer X ligand, a trend that correlates well with aurophilicity. This result also suggests that electronic perturbation caused by Au(I)-Au(I) interactions is important in explaining the reactivity of some dinuclear gold(I) complexes. The crystal structure for *cis*-dppee(Aul)_2_ shows short intramolecular Au(I)-Au(I) interactions of 2.9526 (6) A°, while the structure of *trans*-dppee(AuI)_2_ , shows intermolecular Au(I)-Au(I) interactions of 3.2292 (9) A°. The substitution of .As for P results in a ligand, *cis*-diphenylarsinoethylene (*cis*-dpaee), that is photochemically active, in contrast to the *cis*-dppee ligand. The complexes, *cis*-dpaee(AuX)_2_, are also photochemically active but with lower quantum yields than the *cis*-dppee(AuX)_2_ complexes.


### ELECTRONIC STRUCTURE OF DINUCLEAR GOLD(I) COMPLEXES

Janet B. Foley,[1] Stanley E. Gay,[1] Christopher Turmel,[1] Gang Wei,[1] Tong Jiang,[1] Ratnavathany Narayanaswamy,[1] Bruce M. Foxman,[2] Michael J. Vela,[2] Alice E. Bruce,*[1] and Mitchell R. M. Bruce*[1]

[1] University of Maine, Department of Chemistry, Orono, Maine 04469-5706, USA
[2] Brandeis University, Waltham, MA 02454-9110, USA

### ABSTRACT

Cyclic voltammetry (CV) experiments on $LL(AuSR^*)_2$ complexes [LL = diphenylphosphinomethane (dppm), diphenylphosphinopentane (dpppn); R* = *p*-$SC_6H_4CH_3$] show anodic sweeps that broaden by about 25 mV on going from the longer (dpppn) to the shorter (dppm) bidentate phosphine ligand. Changing concentrations had no effect on the shape of the waveform. The result suggests a weak intramolecular metal-metal interaction in $dppm(AuSR^*)_2$ that correlates well with rate acceleration occurring in the reaction of $dppm(AuSR^*)_2$ with organic disulfides. Quantum yields for *cis*-$dppee(AuX)_2$ [dppee = 1,2-bis(diphenylphosphino)ethylene; X = Cl, Br, I] complexes, $\Phi_{(disappearance)}$, are significantly higher in complexes with a softer X ligand, a trend that correlates well with aurophilicity. This result also suggests that electronic perturbation caused by Au(I)-Au(I) interactions is important in explaining the reactivity of some dinuclear gold(I) complexes. The crystal structure for *cis*-$dppee(AuI)_2$ shows short intramolecular Au(I)-Au(I) interactions of 2.9526 (6) Å, while the structure of *trans*-$dppee(AuI)_2$, shows intermolecular Au(I)-Au(I) interactions of 3.2292 (9) Å. The substitution of As for P results in a ligand, *cis*-diphenylarsinoethylene (*cis*-dpaee), that is photochemically active, in contrast to the *cis*-dppee ligand. The complexes, *cis*-$dpaee(AuX)_2$, are also photochemically active but with lower quantum yields than the *cis*-$dppee(AuX)_2$ complexes.

### INTRODUCTION

The prevalence of gold-gold interactions in solid state structures of gold complexes has prompted study into the theoretical origins of attraction between $d^{10}$ atoms, the strength and requirements for such interactions, and the identification of chemical systems where gold-gold interactions may be important for understanding electronic structure or reactivity. The first estimate of the strength of a gold-gold interaction was made by Schmidbaur and coworkers who carried out the reaction of a syn-anti phosphineylide with gold which produced a dinuclear gold complex with a syn-syn phosphineylide conformation and a short gold-gold bond in the solid state. The energy barrier between the syn-anti and syn-syn phosphineylide conformations was known to be about 9 kcal/mol, thus leading to an estimate of the strength of the Au(I)-Au(I) interaction.[4] More recently, the gold-gold bond has been measured in solution from variable temperature NMR studies of gold-gold bonded and non-bonded conformations.[5,6] All of these estimates suggest that the strength of a gold-gold interaction is approximately 10 ± 1 kcal/mol.

Our group has been pursuing systems where gold-gold interactions may be important to understanding electronic structure or reactivity. The photochemical isomerization reaction of *cis*-$dppee(AuX)_2$ complexes (eq. 1) suggests an important role for Au(I)-Au(I) bonding in the activation process.[7,8] Likewise, the close proximity of Au(I) atoms appears important in accelerating the reaction of disulfide with dinuclear gold complexes with short bridging phosphines. For example, $dppm(AuSR^*)_2$

(eq. 2), reacts much faster with disulfide than mononuclear gold phosphine complexes (eq. 3) or dinuclear gold complexes with longer bridging phosphines (eq. 2, dppe).[9,10,11]

$$\textit{cis}\text{-dppee}(AuX)_2 \xrightarrow{h\nu} \textit{trans}\text{-dppee}(AuX)_2 \qquad (1)$$

$$(X = Cl, Br, I, SC_6H_4CH_3)$$

$$RSSR + LL(AuSR^*)_2 \longrightarrow R^*SSR^* + LL(AuSR)_2 \qquad (2)$$

$$(LL = \text{dppm, dppe}, R = SC_6H_4Cl, R^* = SC_6H_4CH_3)$$

$$RSSR + 2\,Ph_3PAuSR^* \longrightarrow R^*SSR^* + 2\,Ph_3PAuSR \qquad (3)$$

$$(R = SC_6H_4Cl, R^* = SC_6H_4CH_3)$$

### MATERIALS AND METHODS

**Materials.** Acetonitrile (Burdick & Jackson UV grade) was used as received. The supporting electrolyte, tetra-N-butylammonium hexafluorophosphate (TBAH), was prepared by metathesis of tetra-N-butylammonium bromide (TBABr) and $HPF_6$ in water.[12] TBAH was purified by recrystallization from methylene chloride/ether and dried at 80 °C under vacuum for more than 24 hours. The gold complexes and arsine ligands were prepared according to previously published methods.[5,13] Phosphine ligands were purchased from Strem or Aldrich; *p*-thiocresol was purchased from Aldrich. $^1H$ NMR spectra were recorded on a Varian 300 MHz spectrometer at ambient temperature.

**Abbreviations.** The following abbreviations are used: LL = bis(diphenylphosphino)methane (dppm), 1,2-bis(diphenylphosphino)ethane (dppe), 1,5-bis(diphenylphosphino)pentane (dpppn), 1,2-bis-(diphenylphosphino)ethylene (dppee), 1,2-bis-(diphenylarsino)ethylene (dpaee).

**Cyclic voltammetry (CV) experiments.** CV experiments were conducted by using an EG&G Princeton Applied Research 273 potentiostat/galvanostat. A remote computer controller and the program HEADSTRT (EG&G PAR) were used to acquire and store data from the PAR 273. CV data were then converted to SPECTRA CALC (Galactic Software) format using a series of conversion programs. CV measurements were performed in acetonitrile with 0.1 M TBAH as supporting electrolyte. Fresh solutions containing electrolyte (5 mL) were prepared prior to each CV experiment. Each solution was deoxygenated by purging with nitrogen for 5 minutes. Background cyclic voltammograms were acquired before the addition of gold compound. A platinum disk working electrode, a platinum wire auxiliary electrode, and a saturated potassium calomel reference electrode (S.C.E.) comprised the three-electrode system. The working electrode was polished before each set of experiments with 0.25 μ diamond polish (Metadi) and was wiped clean prior to each measurement. The auxiliary electrode was lightly sanded before each set of experiments with fine sand paper. Potentials are reported vs. S.C.E. at room temperature and are not corrected for junction potentials. All the experiments were repeated several times.

### RESULTS AND DISCUSSION

### Cyclic Voltammetry Experiments.

The observations outlined in the introduction suggest that Au(I) atoms in close proximity perturb electronic structure and reactivity. Since metal-metal interactions have been shown to broaden the shape of cyclic voltammetry waves,[14] oxidative cyclic voltammetry experiments were performed on $LL(AuSR^*)_2$ complexes (LL = dppm, dpppn). Previous electrochemistry investigations on phosphine gold thiolate complexes show the presence of an oxidation process at about +0.6 V (*vs.* SCE).[15,16] These processes involve an irreversible one-electron oxidation followed by a fast chemical reaction. A qualitative inspection of cyclic voltammetry anodic sweeps of $LL(AuSR^*)_2$ complexes shows that when LL is changed from dpppn, to dppe, to dppm, the oxidation wave becomes broader. One quantitative measure of the broadness of a wave, is to measure the difference in potential between peak current and current at half-height. Table 1 shows the results of this analysis for LL = dpppn and dppm at two concentrations. Changing concentration has no effect on the width of the peak. However, the oxidative wave for $dppm(AuSR^*)_2$ is broader than that for $dpppn(AuSR^*)_2$ by about 25 mV. This result suggests that a weak metal-metal interaction occurs in $dppm(AuSR^*)_2$ which correlates well with the rate acceleration observed in the reaction of $dppm(AuSR^*)_2$ with disulfide (eq. 2).

**TABLE 1.** Width of anodic sweep voltammograms ($V_{peak}$-$V_{1/2\ current}$) for $LL(AuSR^*)_2$ using a Pt working electrode at 100 mV/s in 0.1 M $TBAH/CH_3CN$ solution.

| Complex | Concentration | $V_{peak}$-$V_{1/2\ current}$ |
|---|---|---|
|  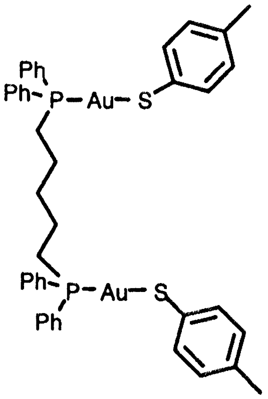 | 1.9 x $10^{-4}$ M | 89 mV |
| | 4.2 x $10^{-4}$ M | 90 mV |
|  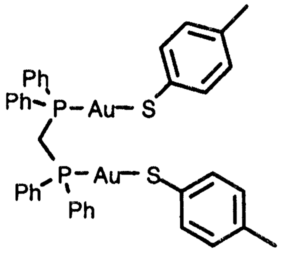 | 2.1 x $10^{-4}$ M | 113 mV |
| | 4.2 x $10^{-4}$ M | 117 mV |

**Reducing the Degrees of Freedom for Rotation.**

The observation that dppm$(AuSR^*)_2$ reacts much faster with disulfide than does dppe$(AuSR^*)_2$, led to attempts to synthesize and characterize *cis*-dppee$(AuSR^*)_2$ (see Figure 1a) which has one degree of freedom less for rotation than dppe$(AuSR^*)_2$. This reduction in freedom for rotation and the cis orientation of the phosphine, results in the Au(I) atoms being held in close proximity. In contrast, *trans*-dppee$(AuSR^*)_2$ also has one less degree of freedom for rotation but results in the Au(I) atoms being held very far apart (see Figure 1b).

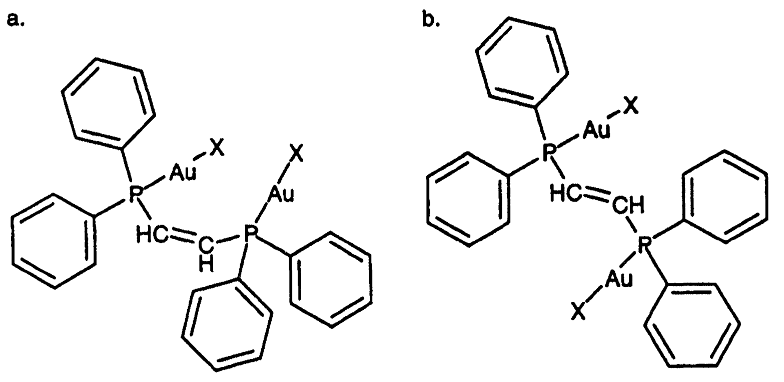

**Figure 1.** The complexes (a)*cis*-dppee$(AuX)_2$ and (b) *trans*-dppee$(AuX)_2$, X = Cl, Br, I, SR*.

Prior to trying the reaction of *cis*-dppee$(AuSR^*)_2$ with disulfide, the syntheses and characterization of *cis*-dppee$(AuX)_2$ and *trans*-dppee$(AuX)_2$ (X = Cl, Br, I, SR*) complexes were first explored. The syntheses and X-ray crystal structures of *cis*-dppee$(AuCl)_2$ and *trans*-dppee$(AuCl)_2$ complexes have been previously reported,[17,18,19,20] as has the synthesis of *cis*-dppee$(AuBr)_2$.[17] While we were able to prepare the chloride and bromide complexes without difficulty, the syntheses of *cis*-dppee$(AuI)_2$ and *cis*-dppee$(AuSR^*)_2$ proved problematic, owing to an impurity that formed during synthesis.[7,21,22] It soon became evident that a significant amount of the *trans*-dppee complexes formed during syntheses and recrystallization, which upon further investigation led to the discovery that a photochemical process was responsible for these transformations (eq 1).[7] Interestingly, the *cis*-dppee ligand is not photochemically reactive nor does it appear that the *trans*-dppee$(AuX)_2$ complexes are photochemically reactive. Thus the experimental results suggest that the close proximity of the gold atoms is critical to the excited state reactivity. Recent *ab intio* calculations also support this view.[8]

Exclusion of light allows high purity preparation of *cis*-dppee$(AuI)_2$ and *trans*-dppee$(AuI)_2$ and recently X-ray analyses were completed.[22] Table 2 compares selected bond lengths and angles for *cis*- and *trans*-dppee$(AuX)_2$ complexes [X = Cl, I].[19,20,22] Unfortunately, the synthesis of *cis*-dppee$(AuSR)_2$ is still somewhat problematic in our hands and thus to date, we have not successfully grown crystals for X-ray analysis nor have we completed a study on the reactivity with disulfide.

**Quantum Yields and Arsine Analogs.** The apparent efficiency in the photochemical isomerization of *cis*-dppee$(AuX)_2$ (X = C, Br, I) complexes,[7] prompted measurement of quantum yields. The results of $\Phi_{(disappearance)}$ studies are summarized in Table 3.[13] The photochemical transformations of *cis*-dppee$(AuX)_2$ to *trans*-dppee$(AuX)_2$ are extremely clean indicating that $\Phi_{(disappearance)}$ is a good approximation of $\Phi_{(cis\text{-}to\text{-}trans)}$. Interestingly, the complexes with a softer X ligand, have significantly higher quantum yields, a trend that correlates well with aurophilicity.[23,24]

**TABLE 2.** Selected bond lengths & angles for *cis*- and *trans*-dppee$(AuX)_2$ complexes [X = Cl, I].

| Complex | Au-Au interaction (Å) [type] | Au-X length (Å) | Au-P length (Å) | P-Au-X (°) | Ref. |
|---|---|---|---|---|---|
| *cis*-dppee$[AuCl]_2$ | 3.043 (1) [intra] | 2.299 (5)<br>2.289 (5) | 2.226 (4)<br>2.239 (5) | 172.5 (2)<br>173.3 (2) | 19 |
| *cis*-dppee$[AuI]_2$ | 2.9526 (6) [intra] | 2.5628 (9)<br>2.5384 (9) | 2.256 (3)<br>2.253 (2) | 174.80 (7)<br>170.37 (7) | 22 |
| *trans*-dppee$[AuCl]_2$ | 7.742 (1) [intra]<br>3.043 (1) [inter] | 2.291 (2) | 2.235 (2) | 173.5 (1) | 20 |
| *trans*-dppee$[AuI]_2$ | 6.841 [intra]<br>3.2292 (9) [inter] | 2.5434 (9) | 2.249 (2) | 174.81 (7) | 22 |

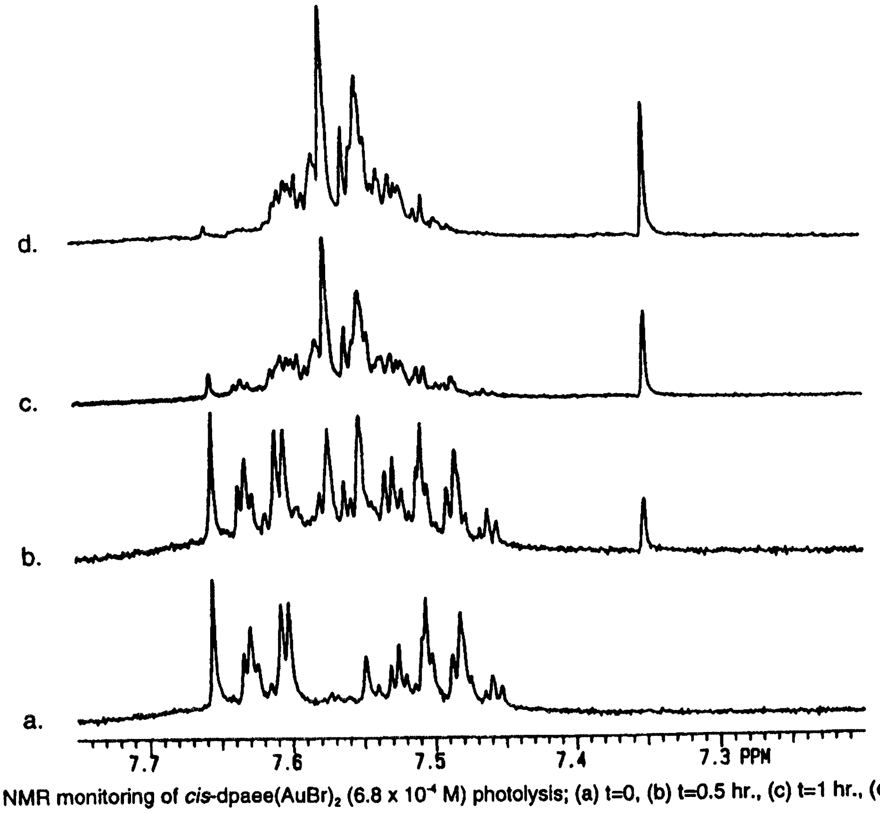

**FIGURE 2.** $^1H$ NMR monitoring of *cis*-dpaee$(AuBr)_2$ (6.8 x $10^{-4}$ M) photolysis; (a) t=0, (b) t=0.5 hr., (c) t=1 hr., (d) t=1.5 hr.

Irradiation of *cis*-dppee$(AuX)_2$ complexes results in an electronic transition that involves a X-to-Au charge transfer transition with a significant contribution of olefin $\pi^*$ character in the LUMO.[7,8] This prompted investigation of the effect of changing P to As in the photochemical isomerization reaction to explore the effect that the linker, X-Au-P-C=C *vs.* X-Au-As-C=C, has on the overall photochemical process.[25] The *cis*-dppee$(AuX)_2$ complexes also undergo photochemical isomerization to *trans*-dpaee$(AuX)_2$. The photochemical transformation of *cis*-dpaee$(AuBr)_2$, monitored by $^1H$ NMR, is shown in Figure 2. The singlet at ca. 7.66 ppm in Figure 2a is assigned to the ethylene protons on the arsine ligand. As photolysis proceeds, this singlet decreases and the singlet at ca. 7.35 ppm, due to the trans isomer grows in. The clean conversion of cis-dpaee(AuBr)$_2$ to trans-dpaee(AuBr)$_2$ occurs over a period of 1.5 hr. using broad band UV-visible irradiation. As shown in Table 3, there is a significant decrease in the quantum yield of disappearance for the arsine complexes. The reason for this large decrease is unclear, but may involve the fact that arsine complexes are in general less stable than phosphine analogs.[25] Another interesting photochemical difference is that the dpaee ligand is photochemically active in contrast to the phosphine analog.

**TABLE 3**. Quantum Yields ($\phi$) for cis-dppee(AuX)$_2$ and cis-dpaee(AuX)$_2$ Complexes (X = Cl, Br, I)

| Complex | $\phi_{(disappearance)}$ | $\lambda$ (nm) | n (replications) | Ref. |
|---|---|---|---|---|
| cis-dppee(AuCl)$_2$ | 0.20 ± 0.06 | 334 | 4 | 22 |
| cis-dppee(AuBr)$_2$ | 0.27 ± 0.09 | 334 | 6 | 22 |
| cis-dppee(AuI)$_2$ | 0.36 ± 0.06 | 334 | 4 | 22 |
| cis-dppee(AuI)$_2$ | 0.37 ± 0.05 | 366 | 3 | 22 |
| cis-dpaee(AuCl)$_2$ | 0.100 ± 0.002 | 313 | 4 | 25 |
| cis-dpaee(AuBr)$_2$ | 0.10 ± 0.01 | 313 | 4 | 25 |

ACKNOWLEDGEMENTS
Whitney King is gratefully acknowledged for assistance in the photochemical quantum yield studies. Johnson Matthey is gratefully acknowledged for a generous loan of hydrogen tetrachloroaurate.

REFERENCES

1. University of Maine, Department of Chemistry, Orono, Maine 04469-5706
2. Current address: Bennington College, Bennington, Vermont, 05201
3. Brandeis University, Waltham, MA 02454-9110
4. Schmidbaur, H . *Gold Bull.*, **23**, 11 -20 (1990).
5. Narayanaswamy, R.; Young M. A.; Parkhurst, E.; Ouellette, M.; Kerr, M. E.; Ho, D.; Elder, R. C.; Bruce, A. E.; Bruce, M. R. M. *Inorg. Chem.*, **32**, 2506-2517 (1993).
6. Harwell, D. E.; Mortimer, M. D.; Knobler, C. B.; Anet, F. A. L.; Hawthorne, M. F., *J. Am. Chem. Soc.*, **118**, 2679-2685 (1996).
7. Foley, J. B.; Bruce, A. E.; Bruce, M. R. M. *J. Am. Chem. Soc.*, **117**, 9596-9597 (1995).
8. Schwerdffeger, P.; Bruce, A. E.; Bruce, M.R.M. *J. Am. Chem. Soc.*, **120**, 6587-6595 (1998).
9. Foley, J.; Fort, R. C., Jr.; McDougal, K.; Bruce, M. R. M.; Bruce, A. E. *Metal-Based Drugs*, **1**, 405-417.
10. DiLorenzo, M. A.; Ganesh, S.; Tadayon, L.; Bruce, M. R. M.; Bruce, A. E., *unpublishedresults*.
11. DiLorenzo, M. A.; Ganesh, S.; Tadayon, L.; Bruce, M. R. M.; Bruce, A. E., *Metal-Based Drugs*, submitted.
12. Bruce, M. R. M.; Megehee, E.; Sullivan, B. P.; Thorp, H.H., O'Toole, T. R.; Downard, A.; Pugh, J. R.; Meyer, T. *J. Inorg. Chem.*, **31**, 4864-4873 (1992).
13. Gay, S. Photochemical Isomerization of Gold(I) Phosphine and Gold(I) Arsine Complexes, University of Maine, M.S. thesis, 1998, and references therein.
14. Downard, A. J.; Honey, G. E.; Phillips, L. F.; Steel, P. *J. Inorg. Chem.*, **30**, 2259-2260 (1991).
15. Jiang, T., Wei, G.; Turmel, C.; Bruce, A. E.; Bruce, M. R. M. *Metal-Based Drugs*, **1**, 419-431 (1994).
16. Mohamed, A. A.; Bruce, A. E.; Bruce, M. R. M. in Organic Derivatives of Silverand Gold, Patai, S., Ed., John Wiley & Sons, England, in press.
17. McAuliffe, C. A.; Parish, R. V.; Randall, P. D. J. Chem.Soc., Dalton Trans, 1730-1735 (1979).
18. Eggleston, D. R.; Chodosh, D. F.; Girrard, G. R.; Hill D. T. Inorg. Chim. Acta., 108, 221-226 (1985).
19. Jones, P. G. Acta Cryst., B36, 2775-2776 (1980).
20. Eggleston, D. S.; McArdle, J. V.; Zuber, G. E. *J. Chem. Soc., Dalton Trans.* 677-679 (1987).
21. Foley, J. B. Chemical and Photochemical Reactivity of Gold(I) Dinuclear Complexes, University of Maine, Ph.D. thesis, 1996, and references therein.
22. Foley, J. B.; Gay, S.; Bruce, A. E.; Bruce, M. R. M.; Foxman, B.; Vela, M. J. unpublished results.
23. Pyykko, P. *Chem. Rev.*, **97**, 597-636 (1997).
24. Schmidbaur, H. *Gold Bull.*, **23**, 11-20 (1990).
25. Gay, S.; Foley, J.; Bruce, A. E.; Bruce, M. R. M. unpublished results.
26. One example of this is PCls, which is quite stable, yet AsCls has never been prepared. See Porterfield, W. W. Inorganic Chemistry, 2nd ed., Academic Press, San Diego, 387-388 (1993).

**Received: December 14, 1998 -**
**Accepted in final form: February 19, 1999**

